# The Proper Treatment of Tubal Gestation

**Published:** 1908-10-31

**Authors:** 


### Gynæcology.

### THE PROPER TREATMENT OF TUBAL GESTATION.

It is barely twenty-five years since Lawson Tait performed the first laparotomy for a fully-ruptured tubal pregnancy, though prior to that the operation had been suggested as an appropriate proceeding in cases of severe pelvic hæmorrhage. At that time the detection of a tubal gestation sac before rupture was supposed to be impossible, and five years passed before an American gynæcologist, Dr. Janvrin, recommended the removal of the unruptured tube as soon as the diagnosis of gestation therein should be made. Since then the free and universal adoption of surgical measures has illuminated many questions previously obscure: thus it is only of late years that this condition has been recognised as almost the sole cause of pelvic hæmatocele.

Whenever a new surgical principle attains striking success and recognition there are at first always two classes of extremists: those who push it to excess, and those who trust it too little. In the case of tubal gestation the latter element is by this time almost eliminated, though for some time it was much in evidence; but opinions still differ as to where the safe and middle course lies. The American Gynæcological Society has lately held a symposium on this question, whereto leading specialists contributed most instructive papers. On some points unanimity prevailed, on others something less; and as the question is one upon which differences of opinion are notorious in Britain also, it is worth while noting some of the points raised in the discussion.

Several of the authors of communications have had personal experience of over one hundred cases apiece, and there are certain matters on which these gentlemen are agreed. They regard the proportion of cases of dangerous hæmorrhage following the rupture of a tubal gestation as about 5 per cent. of the whole number: on this there is little difference of opinion, but on the correct treatment of these patients there is very considerable divergence. The difficulty which everyone admits in this sort of case is to decide whether immediate operation, with its additional shock, shall be imposed upon a woman already in a condition of serious collapse from internal hæmorrhage. On the one side it is argued that even among this 5 per cent. deaths from the initial hæmorrhage are almost unknown; on the other that the correct surgical treatment of profuse and dangerous hæmorrhage is immediate ligature of the bleeding-point. The more conservative school says: wait a few hours until by absolute rest and, if necessary, opium, the patient is so far recovered as to stand the operation. The more advanced teaches: Open the abdomen at once, tie the bleeding spot, remove by hand any large clots, and sew up without a moment's loss of time; remove the embryo if it presents itself, but whatever else you do, be quick. Both sides are opposed to saline infusion, stimulants, or anything else which will increase the blood-pressure, previously, that is, to ligature of the bleeding-point. The question is a thorny one even for skilled and experienced pelvic specialists: probably in general practice when out of reach of expert assistance the balance of opinion will be in favour of operating later rather than at once; that is, after six, twelve, or twenty-four hours.

As to the treatment of the remaining 95 per cent. of tubal ruptures or tubal abortions, there is more agreement. It is admitted that there are considerable advantages to be gained by delaying operation in these cases, provided facilities are existent for immediate laparotomy should symptoms quickly recur. It is also common ground that operative treatment is necessary in the great majority of these patients, and the only question is whether it should be carried out as soon as the conditions—of assistance, surroundings, patient's condition, asepsis, and so on—become reasonably favourable, or whether further symptoms should be awaited. It is undeniable that a certain number of patients who exhibit symptoms and signs of tubal rupture or abortion do recover absolutely without any treatment at all, and the point at issue is whether these women constitute an appreciable proportion or only an insignificant one of the whole number.

The preponderance of opinion among American gynæcologists, as in this country, is in favour of operation and against expectant treatment; but, at the same time, it must be recognised that finality is not yet arrived at. Our text-books certainly ordain operation as a necessary corollary of the diagnosis, or even of the reasonable suspicion, of a tubal pregnancy; but there are a certain number of gynæcologists of eminence who feel convinced that the proportion of spontaneous recoveries has been underestimated, and that a waiting game is often justifiable and legitimate. According to this view, it is right and proper, if preparations are made for instant laparotomy in case of need, to watch a patient who has exhibited signs of a leaking or partially-ruptured tube, or of a tubal abortion, and to order meanwhile absolute rest and non-stimulating regimen. Moreover, *a fortiori*, the same holds good for a gravid tube which has caused nothing suggestive of hæmorrhage. It is believed by those who advocate this line of treatment that many tubal gestation sacs are converted into harmless moles, that a certain number of tubal abortions settle down without ever threatening danger, and that even of actual ruptures some will be found to need no treatment.

It will thus be seen that the time has scarcely come to formulate hard and fast rules for the exact treatment of ectopic gestation: for teachers are not yet unanimous about it. Before leaving the question it may be useful to call attention to two points on which there will be little difference of opinion. One is that in all these cases pelvic examination is to be conducted with the utmost gentleness and care, and is to be avoided except when absolutely necessary. The other is that considerable risk is incurred in the transport of a patient who has had a bleeding tube from her residence to a hospital or nursing home. Unless this can be easily accomplished with the very minimum of disturbance, it may be wiser to face the difficulties of a laparotomy in a private house, or even a tenement.

